# PD-L1 Negative Advanced Non-Small Cell Lung Cancer: Practice Patterns and Real-World Outcomes

**DOI:** 10.3390/curroncol33030144

**Published:** 2026-02-28

**Authors:** Audrey-Ann Bégin, Maude Dubé-Pelletier, Catherine Labbé, Vicky Mai, Michaël Maranda-Robitaille, Marie-Hélène Denault

**Affiliations:** Centre de Recherche de l’Institut Universitaire de Cardiologie et de Pneumologie de Québec (CRIUCPQ), Université Laval, 2725 Ch Ste-Foy, Quebec, QC G1V 4G5, Canada

**Keywords:** metastatic non-small cell lung cancer, PD-L1 negative, chemo-immunotherapy, immune checkpoint inhibitors, real-world evidence

## Abstract

The standard first-line treatment for metastatic non-small cell lung cancer (NSCLC) without oncogenic alterations and programmed death-ligand 1 (PD-L1) expression < 1% is a combination of chemotherapy (CT) and immunotherapy (IO). However, real-world overall survival (OS) appears more modest than in clinical trials, averaging 10–13 months. This retrospective study aimed to assess treatment patterns and real-world outcomes at the Institut universitaire de cardiologie et de pneumologie de Québec (IUCPQ). Patients diagnosed between January 2019 and December 2023 with advanced PD-L1 <1% NSCLC and treated with palliative intent at IUCPQ were included and categorized by first-line treatment. Progression-free survival (PFS) and OS of the CT + IO and CT groups were compared using Kaplan–Meier curves and Cox regression analyses. Data regarding regimen selection, adverse events and subsequent treatment lines were collected. Among 217 eligible patients, 82 (37.8%) received CT + IO, 32 (14.7%) CT alone, 16 (7.4%) targeted therapy, and 87 (40.1%) supportive care. Median PFS was 5.3 vs. 4.7 months (*p* = 0.5) and OS 14.4 vs. 13.5 months (*p* = 0.2) for CT + IO and CT alone, respectively. In the CT + IO group, treatment discontinuation was mainly due to disease progression (59.4%) or adverse events (36.2%). Immune-related adverse events occurred in 29.3%, most frequently pneumonitis (8.5%). Therefore, in this cohort, no statistically significant survival difference was observed between CT + IO and CT alone. However, these findings should be interpreted cautiously given the non-randomized design, baseline imbalances between groups, and the limited sample size of the CT alone cohort. Tolerability of CT + IO was consistent with that observed in clinical trials.

## Simple Summary

For patients with advanced non-small cell lung cancer whose tumors do not express PD-L1, treatment often combines chemotherapy with immunotherapy. Although clinical trials suggest this approach can improve survival compared to chemotherapy alone, outcomes appear more modest in the real-world setting. We reviewed practice patterns and survival outcomes of patients treated at a Canadian academic center over five years. We found that, in our cohort, adding immunotherapy to chemotherapy did not significantly improve survival compared with chemotherapy alone, likely due to insufficient statistical power. However, tolerance to treatment was similar to that reported in clinical trials. The limited number of patients in our study, the broader eligibility criteria used in real-world practice, and the fact that some medically fit patients declined combination treatment may explain these results. These findings suggest that clinical trial results may not fully reflect real-world populations and highlight the need for better tools to support treatment decisions and patient discussions.

## 1. Introduction

In Canada, it is estimated that one in fourteen people will develop lung cancer, which accounts for 25% of all cancer-related deaths—making it the deadliest form of cancer in the country [1]. In the last few years, several new treatments have become available to improve outcomes for patients with metastatic non-small cell lung cancer (NSCLC). Immunotherapy (IO), alone or in combination with chemotherapy (CT), has become the standard of care for the first-line treatment of NSCLC without a readily targetable oncogenic alteration, improving overall survival (OS) compared to CT alone [2,3,4,5,6,7,8,9]. The KEYNOTE-407 (KN407) and KEYNOTE-189 (KN189) studies (pembrolizumab + CT), in addition to the CheckMate-9LA (CM9LA) study (nivolumab + ipilimumab + CT), are some of the key clinical trials that have brought these treatments into daily practice [2,3,4]. However, the addition of IO to CT does not seem to benefit all PD-L1 subgroups equally. In the KN189, KN407 and EMPOWER-Lung 3 studies, it improved OS in patients with PD-L1 < 1%, ranging from 13 to 17 months, but to a lesser extent compared to patients with PD-L1 ≥ 1% [2,3,5]. In contrast, the CM9LA study, which used a distinctive dual IO regimen combined with two cycles of chemotherapy, suggested that patients with PD-L1 < 1% tumors benefited more from the addition of dual IO than those with higher PD-L1 expression (≥1%) [4]. These differing subgroup results, derived from trials not powered for PD-L1–stratified comparisons, contribute to ongoing clinical uncertainty regarding the true magnitude of benefit of chemo-immunotherapy in PD-L1–negative disease. Furthermore, OS tends to be shorter in the real-world setting, underlining a selection bias inherent to clinical trials [10,11]. Indeed, recent studies have observed a real-word overall survival (rwOS) of about 10 to 13 months in the PD-L1 negative metastatic NSCLC (mNSCLC) cohort treated with CT + IO [12,13,14]. Real-world evidence balancing benefits and risks of this combination in this population, however, remains scarce. Given the lack of a known biomarker to further refine patient selection for combination therapy in this subgroup, and the potential for increased toxicity, better characterization of real-world effectiveness and tolerability is clinically relevant. The objective of this study was to assess treatment decisions, practice patterns and real-world outcomes of PD-L1 < 1% advanced NSCLC in our academic center.

## 2. Materials and Methods

### 2.1. Study Design and Population

This study was a single-center retrospective cohort study conducted at the Institut universitaire de cardiologie et de pneumologie de Québec (IUCPQ), an academic center offering tertiary level care in the province of Quebec, Canada. PD-L1-negative patients were identified through pathology records. Patients diagnosed between January 2019 and December 2023 with PD-L1-negative (<1%) advanced NSCLC who were treated with palliative intent were included. Patients treated in other centers were excluded unless clear follow-up documentation was available. The cut-off date for data collection was 31 August 2024.

### 2.2. Data Collection

Data were collected manually through patients’ electronic health records. Variables included age at diagnosis, sex, tobacco exposure, performance status (PS) prior to treatment initiation according to the Eastern Cooperative Oncology Group (ECOG) scale, histology, mutational status, stage, information about first and second lines of treatment, toxicity, disease progression as well as date and cause of death. The study population was then divided into four groups according to first-line treatment: CT + IO, CT alone, targeted therapy and supportive care.

### 2.3. Outcomes

Primary outcomes were real-world OS and PFS. OS was defined as the time from treatment initiation to death from any cause. PFS was defined as the time from treatment initiation to documented disease progression on imaging, as reported by the radiologist, or death. Secondary outcomes included reasons for not choosing CT + IO as first-line treatment, reasons for treatment discontinuation and eligibility for second-line treatment. We also looked at clinically significant treatment-related adverse events (TRAEs), i.e., that led to a delay or discontinuation of treatment, required a dose reduction, resulted in hospitalization or death, or required systemic therapy. Among those, we collected immune-related adverse events (irAEs) for the CT + IO group.

### 2.4. Statistics

Descriptive statistics were used to summarize patient characteristics, first-line treatment, practice patterns and toxicity. Chi-square tests were also performed to identify statistically significant differences between groups, where applicable. For OS and PFS, Kaplan–Meier survival curves were generated for the CT + IO and CT groups, and an exploratory subgroup analysis was conducted based on histological subtype (squamous vs. nonsquamous). Participants were analyzed based on the first-line treatment they received, following an as-treated approach. Patients with missing ECOG PS were included in the survival analyses, but a sensitivity analysis excluding these patients was conducted. Prognostic factors were evaluated using Cox regression. The following variables were first tested in univariate analyses: age, sex, smoking history, ECOG PS, stage, histology, presence of brain metastases at diagnosis, and type of platinum. Patients with missing data were excluded from the given analysis. Variables that were significant in univariate analyses (*p* < 0.1) were included in multivariate analyses. The final model included variables with a *p*-value < 0.05. The threshold for statistical significance was set at *p* < 0.05 for all the other analyses.

## 3. Results

### 3.1. Patients and Treatments

**Figure 1:**
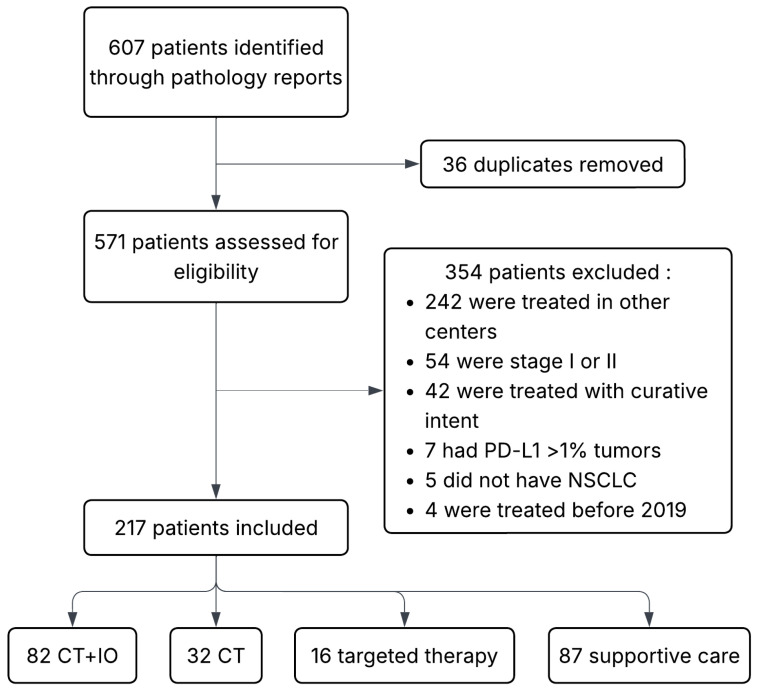
Summary of patient selection from pathology reports dating from January 2019 to December 2023. NSCLC indicates non-small cell lung cancer; PD-L1, programmed death-ligand 1; CT + IO, chemotherapy and immunotherapy; CT, chemotherapy.

A total of 217 patients were included in this study (Figure 1). Eighty-two (37.8%) were treated with CT + IO, 32 (14.7%) with CT alone, 16 (7.4%) with targeted therapies and 87 (40.1%) with supportive care. Most patients had previous tobacco exposure and an ECOG PS of 0 or 1. One hundred and seventy-six (81.1%) had nonsquamous NSCLC, most of which had no oncogenic alterations. A minority (12.4%) had cerebral metastases at diagnosis. In the CT-alone group, there was a higher proportion of men, squamous histology and ECOG 2, but fewer patients had brain metastases (Table 1).

The main reasons for not choosing CT + IO were poor PS (33.3%), patient’s decision (24.4%), ineligibility according to physician’s evaluation (19.3%) and targeted therapy initiated instead (12.6%) (Table 2). Only nine (6.7%) patients had a contraindication, in the treating physician’s opinion, to immunotherapy. While most patients had appropriate PS at diagnosis to receive CT + IO (ECOG 0–1), some were eventually deemed unfit because of clinical deterioration before treatment initiation. Furthermore, for 16 patients (19.5%), PS at the beginning of CT + IO was not documented in the chart. The median time interval between tissue sampling and treatment initiation was 25 days (interquartile range [IQR] 18–34) for CT + IO and 23 days (IQR 15–47) for CT.

In the CT + IO group, all patients received a platinum doublet combined with pembrolizumab. Most patients (90%) received carboplatin in both groups (CT + IO and CT alone). Fifty-two (63.4%) patients treated with CT + IO completed four cycles of the triplet therapy. Sixty-nine (84.1%) patients treated with CT + IO and 23 (71.9%) treated with CT alone discontinued treatment, mostly because of disease progression (59.4% vs. 60.9%), followed by TRAEs (36.2% vs. 30.4%) (Table 3).

### 3.2. Survival Outcomes

**Figure 2:**
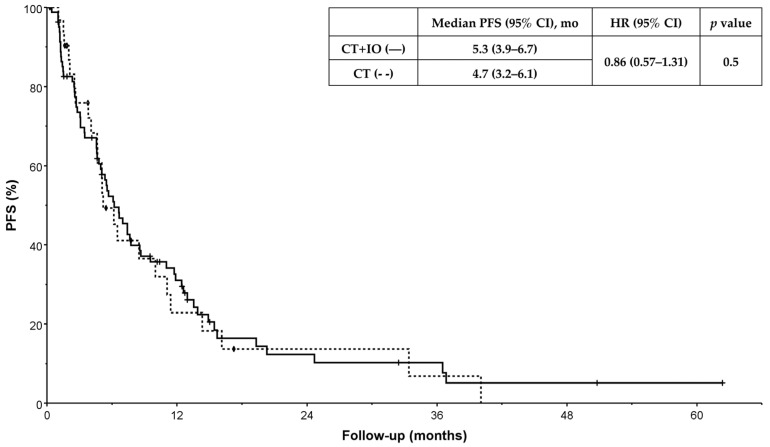
Progression-free survival (PFS) according to first-line treatment. PFS was defined as the time from treatment initiation to documented disease progression on imaging or death. Date of data cut-off was 31 August 2024. CT + IO indicates chemotherapy and immunotherapy; CT, chemotherapy; HR, hazard ratio.

**Figure 3:**
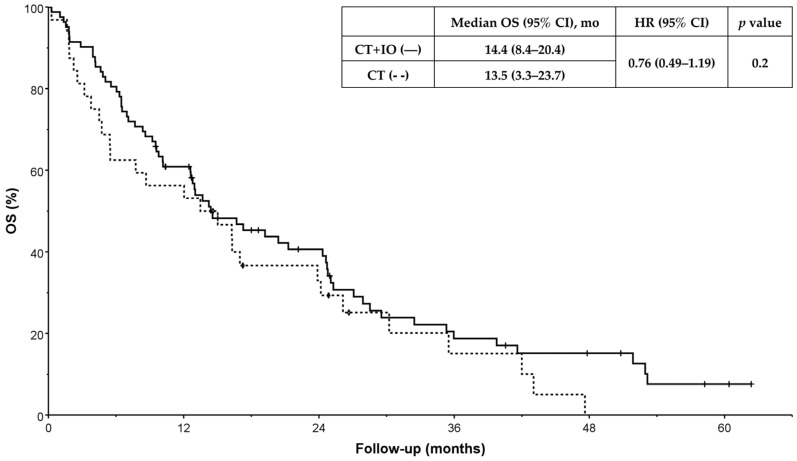
Overall survival (OS) according to first-line treatment. OS was defined as the time from treatment initiation to patient’s death. Date of data cut-off was 31 August 2024. CT + IO indicates chemotherapy and immunotherapy; CT, chemotherapy; HR, hazard ratio.

At the time of data cut-off, 64 deaths had occurred in the CT + IO cohort, accounting for 78.0% of patients in this group, and 75 (91.5%) had progressed. In the CT-alone cohort, 28 (87.5%) deaths had occurred, and 31 (96.9%) patients had progressed. There were no significant differences in OS (14.4 vs. 13.5 months, HR = 0.76 [95%CI: 0.49–1.19], *p* = 0.2) or PFS (5.3 vs. 4.7 months, HR = 0.86 [95%CI: 0.57–1.31], *p* = 0.5) between CT + IO and CT alone (Figure 2 and Figure 3). Excluding patients with missing ECOG performance status, median PFS was 6.1 versus 4.7 months (HR = 0.84 [95%CI: 0.54–1.32], *p* = 0.4), and median OS was 16.7 versus 13.5 months (HR = 0.72 [95%CI: 0.45–1.17], *p* = 0.2).

In the squamous NSCLC subgroup, median OS was 6.5 months with CT + IO versus 16.3 months with CT alone (*p* = 1.0), and median PFS was 5.5 versus 5.0 months (*p* = 0.4) (Figures S1 and S2). In the nonsquamous subgroup, median OS was 14.6 months with CT + IO versus 7.7 months with CT alone (*p* = 0.1), and median PFS was 5.0 versus 4.1 months (*p* = 0.8) (Figures S3 and S4). Among patients in the squamous subgroup initially treated with CT alone, seven received nivolumab as second-line treatment. These patients had the longest OS within the subgroup, ranging from 16.3 to 47.6 months. Their second progression-free survival (PFS2)—measured from first-line treatment initiation to radiologic progression under second-line treatment—ranged from 6.6 to 26.7 months, with a median of 20.4 months.

Stage IVB (versus stages III and IVA) and brain metastases at diagnosis significantly decreased overall survival (HR = 2.68 [95%CI: 1.31–5.50], *p* = 0.01 and HR = 1.99 [95%CI: 1.10–3.59], *p* = 0.02, respectively) and were thus tested in a multivariate model including the type of first-line treatment. Stage IVB remained significant (HR = 2.65 [95%CI: 1.28–5.50], *p* = 0.01), whereas the type of first-line treatment and brain metastases remained non-significant (*p* = 0.11 and *p* = 0.09, respectively).

A similar proportion of patients underwent second-line treatment (53.7% vs. 50.0%, *p* = 0.7), which mostly consisted of chemotherapy (88.6%) in the CT + IO group and immunotherapy (68.8%) in the CT-alone group (Table 4).

### 3.3. Toxicity

In patients treated with CT + IO, 44 (53.7%) patients experienced clinically significant toxicity, among which 24 (29.3%) patients had clinically significant irAEs. The most frequent TRAEs were hematological (11.0%). Clinically significant immune-related pneumonitis occurred in 8.5% of patients, making it the most frequently reported irAE, followed by rash, colitis and thyroiditis which each occurred in 4.9% of cases. In patients treated with CT alone, 14 (43.8%) experienced clinically significant TRAEs. Most TRAEs were also hematological (18.8%) (Table 5).

## 4. Discussion

This study was not able to demonstrate a significant improvement in OS or PFS with CT + IO compared to CT alone for patients treated at our center. Several baseline characteristics differed between the CT + IO and CT-alone groups, including sex distribution, histology, ECOG performance status, and presence of brain metastases. Differences in chemotherapy backbone between groups may have influenced outcomes as well. These imbalances likely confounded survival comparisons and should be weighed in when interpreting differences in OS and PFS between groups. The CT-alone group included a relatively small number of patients, and a lower proportion of patients had central nervous system involvement or stage IVB disease, which may have resulted in more favorable outcomes for this group. Indeed, stage IVB was negatively associated with survival in the multivariate analyses, supporting this interpretation. There might have been unmeasured favorable prognostic factors in the CT-alone group as well. However, there was a higher proportion of patients with ECOG PS 2 and squamous NSCLC in the CT alone group, both typically associated with worse prognosis, although our univariate analyses did not demonstrate this.

Interestingly, overall survival was longer in the squamous subgroup that received CT alone as first-line therapy followed by nivolumab at progression, compared with those who did not receive nivolumab. These patients also demonstrated longer PFS2, suggesting that patients who responded to nivolumab may have achieved prolonged disease control, as previously shown in second-line immunotherapy trials [15]. However, these results should be interpreted with caution, as the subgroup analysis according to histology was exploratory and underpowered. Furthermore, only 16 patients in the CT-alone cohort received second-line treatment. The small number of patients and the inherent risk of selection and survival bias—given that only patients with adequate performance status and disease control are able to proceed to second-line therapy—limit interpretation in this context. Therefore, these findings should be considered hypothesis-generating at most.

Survival outcomes in both groups may have been influenced by the absence of clear fitness criteria to determine eligibility for combination therapy, e.g., by the decision to administer CT alone to patients who might otherwise have been suitable candidates for CT + IO. Indeed, 26 patients in the CT-alone group had an ECOG PS of 0–1, while only 9 patients did not get CT + IO because of a contraindication to IO. We also observed that many patients initially classified as ECOG PS 0–1 did not receive CT + IO due to alleged inadequate PS, raising the hypothesis that other factors could have influenced the physician’s decision that are not fully captured by the ECOG PS scale. In 16 (19.5%) patients in the CT + IO group, ECOG PS documentation was even missing altogether. Furthermore, the median time from diagnosis to systemic treatment initiation was well within recommended timelines for both groups. However, the IQRs show wider dispersion in the CT-alone group compared to the CT + IO group, suggesting that delays in treatment initiation could have led to clinical deterioration and precluded combination therapy. These findings truly underscore the added value of real-world insight on practice patterns, which clinical trials did not assess.

In our cohort, real-world progression-free survival (rwPFS) and rwOS among patients treated with CT + IO seemed slightly shorter than those reported in clinical trials [2,3]. This expected difference could be explained by the inclusion of patients with an ECOG PS of 2 or unknown, whereas the KN407 and KN189 trials only enrolled patients with an ECOG PS of 0–1. Supporting this hypothesis, a retrospective study by Wallarabenstein et al. evaluated nonsquamous advanced NSCLC patients treated with CT + IO and found that those meeting KN189 trial eligibility had superior outcomes (median PFS 9.2 vs. 4.6 months; median OS 16.5 vs. 6.5 months) compared to ineligible patients, who represented more than half of the cohort [11]. This highlights how more flexible inclusion criteria in real-world practice may contribute to less favorable survival outcomes compared to clinical trial populations. Furthermore, a real-world study by Verschueren et al. similarly showed a gap between real-world and trial-based survival outcomes, partly attributable to differences in patient selection. Inconsistent imaging intervals may also have contributed to discrepancies in PFS, a factor that could have influenced our results as well [12]. Indeed, rwPFS in our study was derived from routine clinical documentation and radiologic reports rather than standardized RECIST-based assessments, which may have introduced variability in progression ascertainment and potential assessment bias. During the induction phase (triplet or doublet therapy), imaging was generally performed every six weeks (i.e., every two cycles) in both treatment groups. However, beyond this initial phase, imaging intervals varied according to maintenance strategy (CT alone, CT + IO, IO alone, or no maintenance), eligibility for second-line therapy, and evolving institutional practices over the study period. This heterogeneity in post-induction imaging schedules may have contributed to interval censoring and imprecision in the determination of progression dates. Such methodological constraints inherent to retrospective real-world analyses may partially explain differences between our findings and those reported in randomized controlled trials.

CT + IO tolerance appeared consistent with that observed in KN189 and KN407. Treatment discontinuation rates were comparable—84.1% in our cohort, versus 86.3% in KN189 and 85.6% in KN407 [2,3]. A higher proportion of patients treated with CT + IO experienced TRAEs compared to those treated with CT alone (53.7% vs. 43.8%), which was expected and consistent with the literature [2,3]. Although approximately one-third of patients in the CT + IO group discontinued treatment due to TRAEs, most were able to receive second-line therapy. This proportion was similar in both groups, reinforcing the fact that CT + IO remains a relatively safe first-line treatment. In our study, 29.3% of patients treated with CT + IO experienced clinically significant irAEs, compared to 27.2% in KN189 and 35.3% in KN407 [2,3]. Pneumonitis occurred in 8.5% of patients treated with CT + IO, which is clinically meaningful and consistent with clinical trials (pneumonitis of any grade: 4.9% in KN189 and 8.3% in KN407) [2,3]. This underscores the need for careful monitoring for immune-related toxicity in the real-world setting. Local practice favored single-agent immunotherapy (pembrolizumab) over dual therapy (nivolumab + ipilimumab), primarily due to concerns about increased toxicity and uncertainty regarding a survival benefit, as well as limited provincial access to the CM9LA regimen during part of the inclusion period of our study.

Interestingly, approximately one in five patients who did not receive CT + IO had declined this option themselves. This may represent a source of selection bias, as patients declining CT + IO could differ systematically from those receiving combination therapy. It also underscores the importance of thoroughly explaining the risks and benefits of CT + IO and addressing patients’ concerns when discussing treatment options. Several decision aids of varying scope and detail have been developed for patients with advanced or metastatic non-small cell lung cancer, including those that address chemo-immunotherapy [16,17,18,19]. Making such tools more accessible in routine practice—for instance, by providing printed copies and adapting them to local contexts (i.e language and drug availability)—could further improve shared decision-making.

One of the main limitations of this study resides in the complexity of data collection. Clinical information was often fragmented across various components of the electronic records and sometimes missing altogether. Of note, 24.4% of patients had undocumented ECOG PS, limiting interpretability and potentially introducing bias in treatment allocation and survival analyses. To assess the robustness of these analyses, a sensitivity analysis excluding patients with missing ECOG performance status was performed, and findings were consistent with the primary analysis. Furthermore, unmeasured or uncaptured prognostic factors could represent a source of confounding bias in this study. The comparison between the two groups was not randomized, and therapeutic choices might have been influenced by clinical factors that were not captured in this study (i.e., frailty, comorbidity or rapid clinical dynamics). As this study was conducted at a single academic center, referral bias may have influenced patient characteristics and treatment patterns, potentially limiting generalizability. Improved documentation of key clinical information about oncologic history could facilitate future research and minimize the risk of bias and incomplete data recovery. Despite these limitations, this study does focus on a clinically understudied population known to derive limited benefit from chemo-immunotherapy and provides real-world evidence from a Canadian cohort regarding survival outcomes, practice patterns and toxicity. In addition, it highlights key clinical aspects that warrant improvement, particularly documentation practices and patient education.

## 5. Conclusions

This retrospective single-center study did not identify a statistically significant improvement in real-world PFS or OS among patients with PD-L1-negative advanced NSCLC treated with CT + IO compared to CT alone. These findings should be interpreted with caution due to the small sample size, baseline imbalances between treatment groups, and limited statistical power, as well as the relatively favorable outcomes observed in the CT-alone cohort. Our results do support the safety and tolerability of CT + IO, with adverse events consistent with those reported in phase III clinical trials. Larger, multicenter real-world studies with careful adjustment for confounding factors are needed to further assess whether CT + IO provides a survival benefit for PD-L1 negative NSCLC in routine clinical practice.

## Figures and Tables

**Table 1:** Baseline characteristics of patients.

	Whole Population (n = 217)	Chemotherapy + Immunotherapy (CT + IO) (n = 82)	Chemotherapy (CT) (n = 32)
Age, years, mean ± SD	69 ± 8	67 ± 7	67 ± 7
Sex, n (%)			
Male	110 (50.7)	41 (50.0)	21 (65.6)
Female	107 (48.3)	41 (50.0)	11 (34.4)
Tobacco exposure, n (%)	203 (93.5)	77 (93.9)	32 (100)
**Performance status, n (%)**			
ECOG PS 0–1	133 (61.3)	65 (79.3)	26 (81.3)
ECOG PS ≥ 2	31 (14.3)	1 (1.2)	4 (12.5)
Unknown	53 (24.4)	16 (19.5)	2 (6.3)
**Histology, n (%)**			
Squamous	41 (18.9)	13 (15.9)	12 (37.5)
Nonsquamous ^1^	176 (81.1)	69 (84.1)	20 (62.5)
**Stage ^2^, n (%)**			
III	25 (11.5)	7 (8.5)	6 (18.8)
IVA	80 (36.9)	31 (37.8)	13 (40.6)
IVB	112 (51.6)	44 (53.7)	13 (40.6)
**Brain metastases at** **diagnosis, n (%)**	27 (12.4)	13 (15.9)	2 (6.3)
**Genomic alterations, n (%)**			
None	175 (80.6)	71 (86.6)	29 (90.6)
EGFR	15 (6.9)	0 (0)	0 (0)
KRAS G12C	10 (4.6)	6 (7.3)	0 (0)
Other KRAS	10 (4.6)	2 (2.4)	3 (9.4)
ROS1	2 (0.9)	0 (0)	0 (0)
BRAF	2 (0.9)	2 (2.4)	0 (0)
MET exon 14 skipping	1 (0.5)	0 (0)	0 (0)
Other	2 (0.9)	1 (1.2)	0 (0)

^1^ Nonsquamous: adenocarcinoma (n = 149/62/17), adenosquamous (n = 3/0/1), other (n = 24/7/2). ^2^ Staging is according to TNM Classification of Malignant Tumors, 8th edition. SD, standard deviation; ECOG PS, Eastern cooperative oncology group performance status.

**Table 2:** First-line treatment.

Type of Treatment, n (%)	Whole Population (n = 217)
Chemotherapy + immunotherapy (CT + IO)	82 (37.8)
Chemotherapy (CT)	32 (14.7)
Targeted therapy	16 (7.4)
Supportive care	87 (40.1)
**Primary Reason for Not Choosing CT + IO, n (%)**	**Patients Who Did Not Receive CT + IO (n = 135)**
Poor performance status	45 (33.3)
Patient’s decision	33 (24.4)
Ineligible according to physician’s evaluation	26 (19.3)
Targeted therapy initiated	17 (12.6)
Contraindication to immunotherapy	9 (6.7)
Before treatment availability	3 (2.2)
Unknown	2 (1.5)

**Table 3:** Treatment disposition.

	CT + IO (n = 82)	CT (n = 32)
Completed triplet, n (%)	52 (63.4)	N/A
Number of platinum-based chemotherapy cycles, median (IQR)	4 (2–4)	4 (1–4)
Number of maintenance chemotherapy cycles for NSQ, median (IQR)	2 (0–6)	0 (0–1)
Number of immunotherapy cycles, median (IQR)	6 (2–12)	N/A
**Type of chemotherapy, n (%)**		
Pemetrexed + platinum	68 (82.9)	15 (46.9)
Paclitaxel + platinum	13 (15.9)	0 (0)
Gemcitabine + platinum	1 (1.2)	14 (43.8)
Vinorelbine	0 (0)	1 (3.1)
Gemcitabine	0 (0)	1 (3.1)
Etoposide + platinum	0 (0)	1 (3.1)
**Type of platinum, n (%)**		
Cisplatin	8 (9.8)	3 (10.0)
Carboplatin	74 (90.2)	27 (90)
**Type of immunotherapy, n (%)**		
Pembrolizumab	82 (100)	N/A
**Discontinued treatment, n (%)**	69 (84.1)	23 (71.9)
Disease progression	41 (59.4)	14 (60.9)
Treatment-related adverse events	25 (36.2)	7 (30.4)
Patient’s decision	0 (0)	2 (8.7)
Other ^1^	3 (4.3)	0 (0)

^1^ Other: 2 had competing medical conditions precluding further treatment; 1 was initially misdiagnosed. IQR, interquartile range; NSQ, nonsquamous.

**Table 4:** Second-line treatment.

	CT + IO (n = 82)	CT (n = 32)	
**Received second-line treatment, n (%)**	44 (53.7)	16 (50.0)	*p* = 0.7
Chemotherapy	39 (88.6)	5 (31.3)	
Immunotherapy	2 (4.5)	11 (68.8)	
Targeted therapy	3 (6.8)	0 (0)	

**Table 5:** Clinically significant treatment-related adverse events (TRAEs).

Type of Adverse Events, n (%)	CT + IO (n = 82)	CT (n = 32)
Hematological	9 (11.0)	6 (18.8)
Respiratory	7 (8.5)	0 (0)
Cutaneous	4 (4.9)	1 (3.1)
Gastrointestinal	5 (6.1)	0 (0)
Endocrine	4 (4.9)	0 (0)
Renal	4 (4.9)	0 (0)
Hepatic	3 (3.7)	0 (0)
Neurological	2 (2.4)	0 (0)
Fatigue	3 (3.7)	7 (21.9)
Other	5 (6.1) ^1^	3 (9.4) ^2^
**Immune-Related Adverse Events (irAEs), n (%)**	**CT + IO (n = 82)**	**N/A**
Pneumonitis	7 (8.5)	
Rash	4 (4.9)	
Colitis	4 (4.9)	
Thyroiditis	4 (4.9)	
Hepatitis	3 (3.7)	
Nephritis	1 (1.2)	
Pancreatitis	1 (1.2)	
Scleroderma	1 (1.2)	

Clinically significant TRAEs were defined as adverse events that led to a delay or discontinuation of treatment, required a dose reduction, resulted in hospitalization or death, or required systemic therapy. Twenty-four patients experienced irAEs, and one of them experienced two different types of irAEs. ^1^ Other: 2 patients had edema, 2 had nausea and 1 developed localized scleroderma secondary to immunotherapy. ^2^ Other: 1 had a hypersensitivity reaction, 1 had inappetence and 1 had hypotension.

## Data Availability

The data presented in this study are available on request from the corresponding author due to privacy and ethical restrictions.

## Supplementary Materials

The following supporting information can be downloaded at: https://www.mdpi.com/article/10.3390/curroncol33030144/s1, Figure S1: Overall survival (OS) according to first-line treatment in the squamous subgroup; Figure S2: Progression-free survival (PFS) according to first-line treatment in the squamous subgroup; Figure S3: Overall survival (OS) according to first-line treatment in the nonsquamous subgroup; Figure S4: Progression-free survival (PFS) according to first-line treatment in the nonsquamous subgroup.

## Abbreviations

The following abbreviations are used in this manuscript:

NSCLC	Non-small cell lung cancer
PD-L1	Programmed death-ligand 1
CT	Chemotherapy
IO	Immunotherapy
OS	Overall survival
IUCPQ	Institut universitaire de cardiologie et de pneumologie de Québec
PFS	Progression-free survival
KN407	KEYNOTE-407
KN189	KEYNOTE-189
CM9LA	CheckMate-9LA
rwOS	Real-world overall survival
mNSCLC	Metastatic non-small cell lung cancer
PS	Performance status
ECOG	Eastern Cooperative Oncology Group
TRAEs	Treatment-related adverse events
irAEs	Immune-related adverse events
IQR	Interquartile range
HR	Hazard ratio
PFS2	Second progression-free survival
rwPFS	Real-world progression-free survival
