# Supplementary material for: PD-L1 Negative Advanced Non-Small Cell Lung Cancer: Practice Patterns and Real-World Outcomes
Source: Curr Oncol. 2026 Feb 28;33(3):144. doi: 10.3390/curroncol33030144 (PMC13024867; doi:10.3390/curroncol33030144)
Supplement: Supplementary file 1 [file curroncol-33-00144-s001.zip › curroncol-4145016-supplementary.pdf]

PD-L1 Negative Advanced Non-Small Cell Lung Cancer: Practice Patterns and Real-World Outcomes — Supplementary Materials

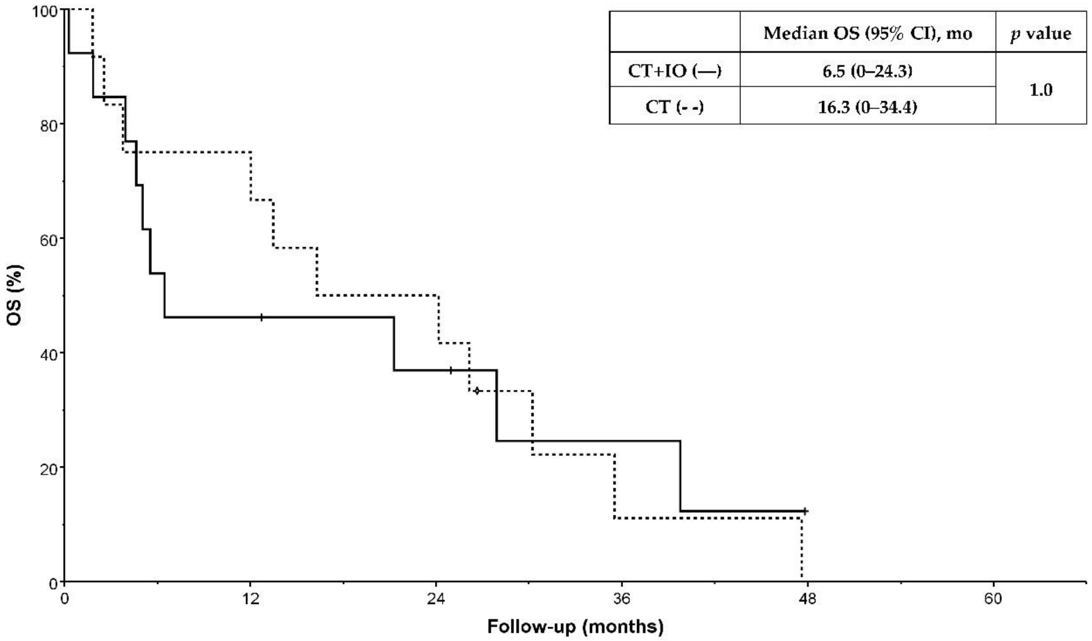

Figure S1. Overall survival (OS) according to first-line treatment in the squamous subgroup. OS was defined as the time from treatment initiation to patient's death. Date of data cutoff was August 31, 2024. CT+IO indicates chemotherapy and immunotherapy; CT, chemotherapy.

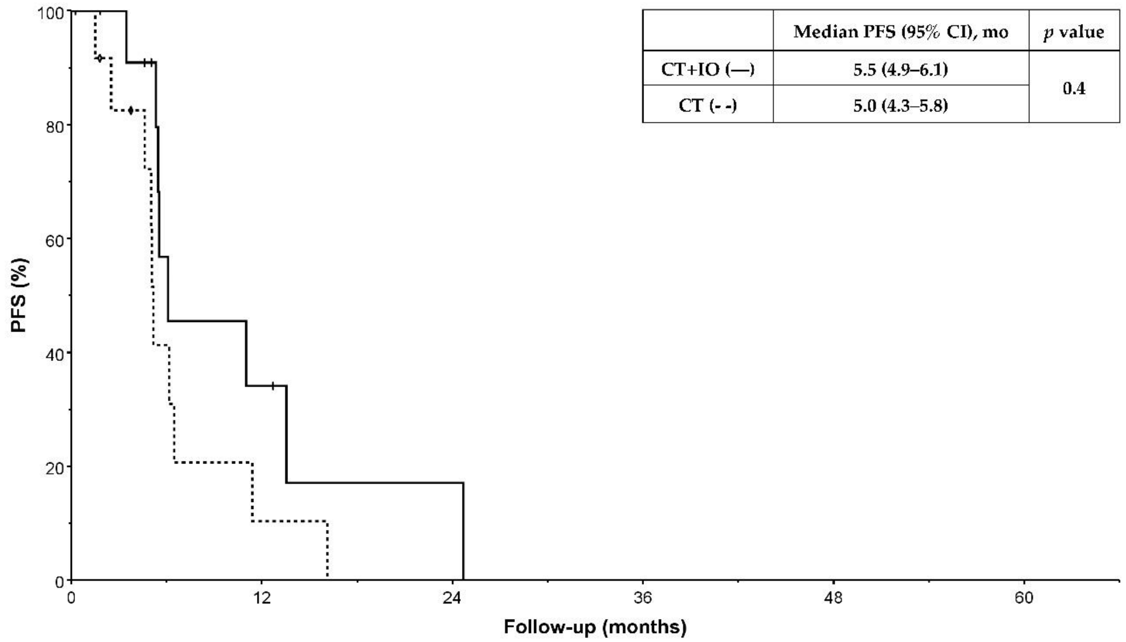

Figure S2. Progression-free survival (PFS) according to first-line treatment in the squamous subgroup. PFS was defined as the time from treatment initiation to documented disease progression on imaging or death. Date of data cutoff was August 31, 2024. CT+IO indicates chemotherapy and immunotherapy; CT, chemotherapy.

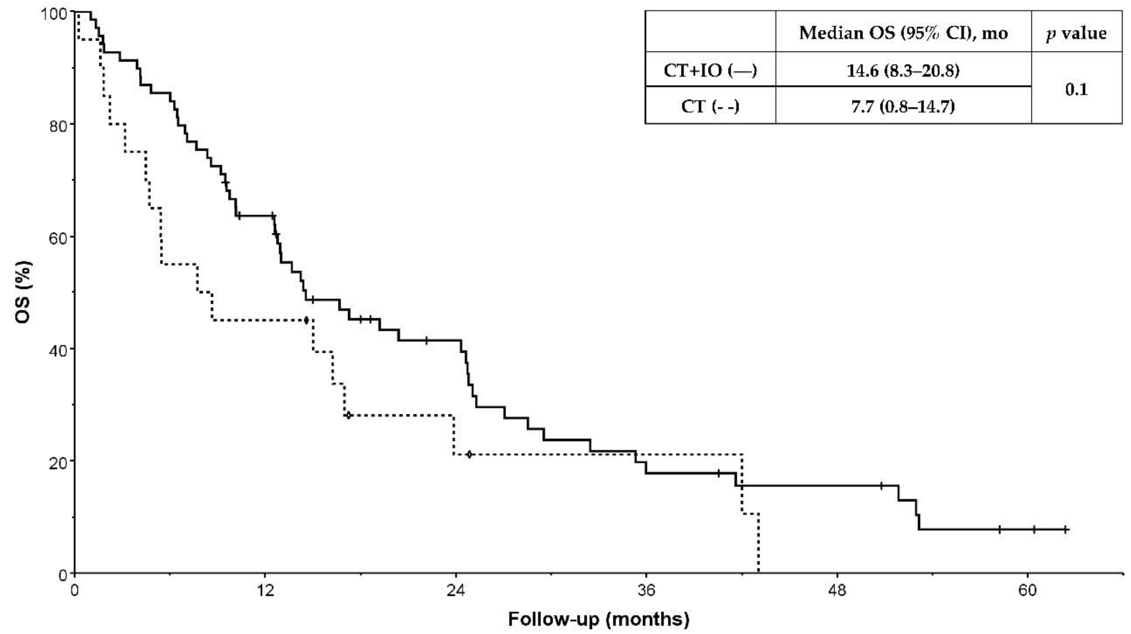

Figure S3. Overall survival (OS) according to first-line treatment in the nonsquamous subgroup. OS was defined as the time from treatment initiation to patient's death. Date of data cutoff was August 31, 2024. CT+IO indicates chemotherapy and immunotherapy; CT, chemotherapy.

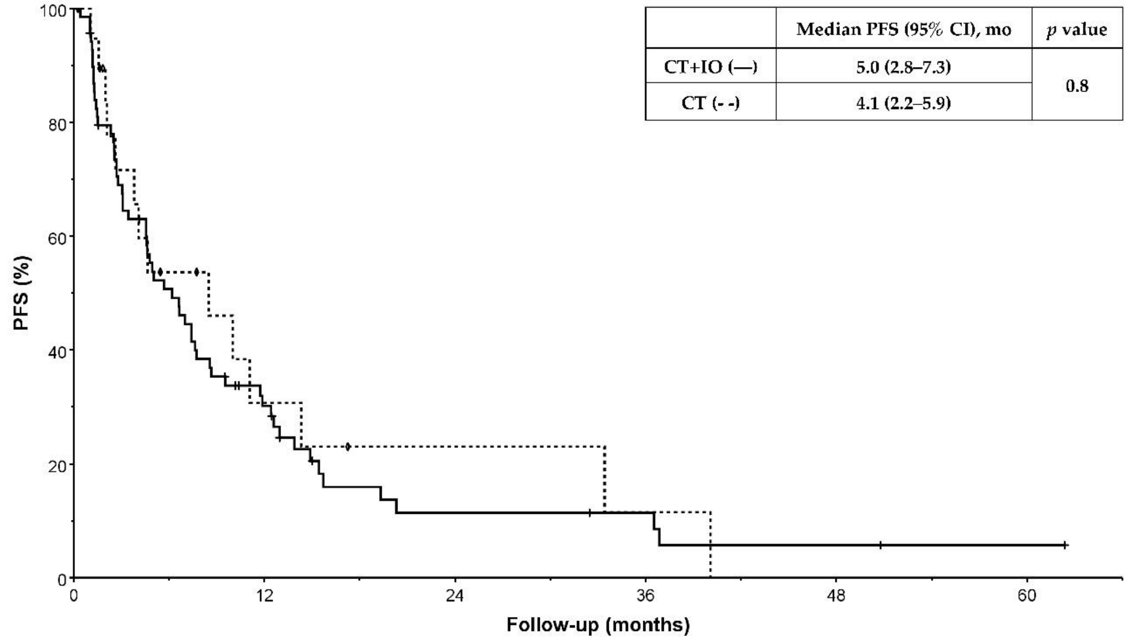

Figure S4. Progression-free survival (PFS) according to first-line treatment in the nonsquamous subgroup. PFS was defined as the time from treatment initiation to documented disease progression on imaging. Date of data cutoff was August 31, 2024. CT+IO indicates chemotherapy and immunotherapy; CT, chemotherapy.
